# Detection and Excretion Profile of Retatrutide in Human Plasma and Urine by LC‐HRMS: Implications for Antidoping Analysis

**DOI:** 10.1002/rcm.70179

**Published:** 2026-09-15

**Authors:** Gabriel Reis Alves Carneiro, Isabelle Karine da Costa Nunes, Gustavo Ramalho dos Santos Cardoso, Paola Façanha dos Santos, Monica Costa Padilha, Fábio César Sousa Nogueira, Henrique Marcelo Gualberto Pereira

**Affiliations:** ^1^ LBCD, LADETEC, Instituto de Química Universidade Federal Do Rio de Janeiro Rio de Janeiro Brazil; ^2^ LABPROT, LADETEC, Instituto de Química Universidade Federal Do Rio de Janeiro Rio de Janeiro Brazil

**Keywords:** antidoping analysis, GLP‐1 receptor agonist, incretin, LC‐HRMS, LY3437943, peptide elimination study, plasma, retatrutide

## Abstract

**Rationale:**

Retatrutide (LY3437943) is a novel long‐acting triple incretin receptor agonist that targets the glucagon‐like peptide‐1 receptor (GLP‐1R), glucose‐dependent insulinotropic polypeptide receptor (GIPR), and glucagon receptor (GCGR). Its pronounced effects on body composition and metabolic health, combined with increasing off‐label use among physically active populations, raise concerns about potential misuse in sport. Despite growing clinical interest and anecdotal reports about use among amateur athletes, information on the detection and elimination profile of retatrutide in humans remains unexplored.

**Methods:**

A method based on liquid chromatography–high‐resolution mass spectrometry (LC‐HRMS) dedicated to monitor retatrutide was developed and validated in human plasma and urine in accordance with World Anti‐Doping Agency (WADA) criteria. Detection was based on the [M + 3H]^3+^ precursor ion by targeted selected‐ion monitoring (t‐SIM), with confirmatory analysis via parallel reaction monitoring (PRM) of structurally diagnostic fragment ions. From four retatrutide users who self‐administered 2 mg subcutaneously on Days 1 and 7, blood and urine samples were collected at predefined time points following self‐administration.

**Results:**

The method was proved fit‐for‐purpose regarding selectivity, reliability, robustness, and stability. Limits of detection (LOD) were evaluated using both empirical ≥ 95% detection rate criteria and sigmoidal logistic regression modeling, yielding 6.14 ng/mL in plasma and 2.44 ng/mL in urine. Neither intact retatrutide nor metabolite‐derived signals were detected in urine samples throughout the entire collection period, despite systematic application of multiple extraction strategies. Plasma concentration–time profiles exhibited nonmonotonic behavior, consistent with prolonged subcutaneous absorption and depot‐like release kinetics following repeated dosing.

**Conclusion:**

These findings suggest that plasma represents the appropriate matrix for intact retatrutide detection compared with urine. The extended plasma detection window, approximately 56 days after the first dose, supports plasma‐based testing as a suitable analytical strategy for this and related long‐acting therapeutic peptides in antidoping testing context.

## 1 Introduction

**FIGURE 1:**
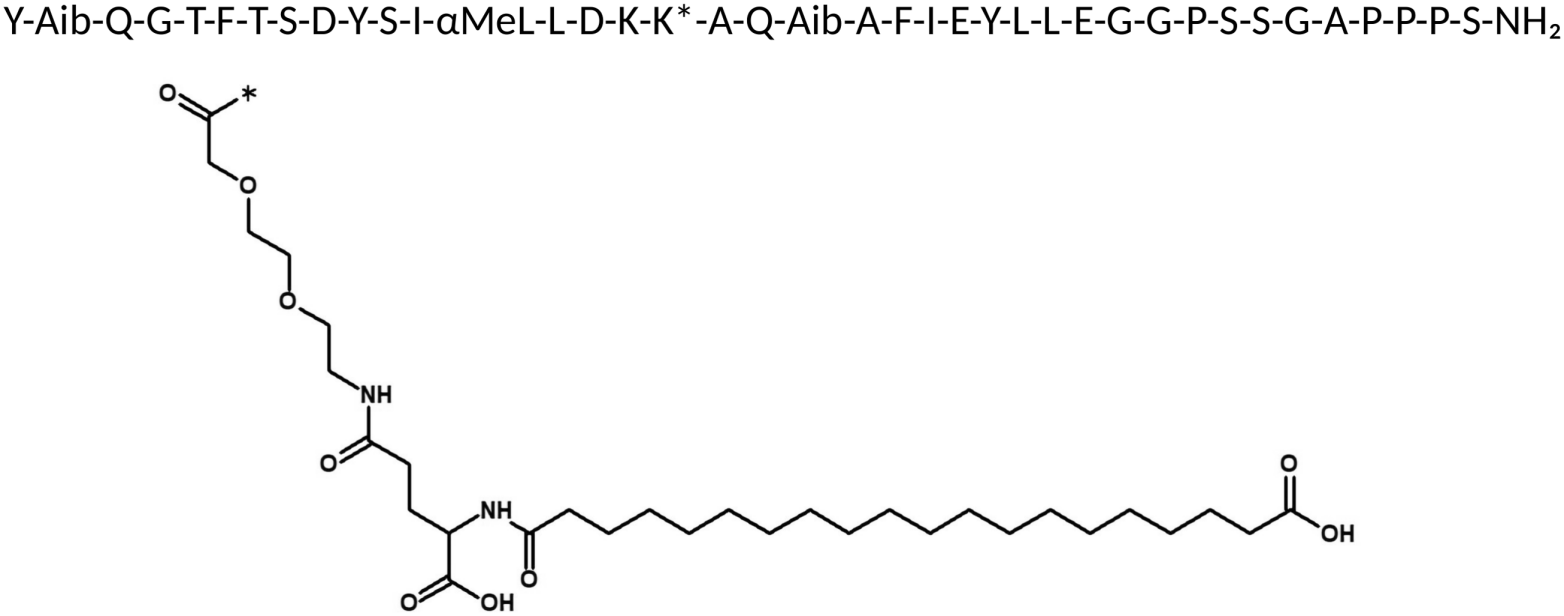
Chemical structure of retatrutide (LY3437943). The 39‐amino‐acid peptide backbone is modified at Lys17 (K17*) by a side chain composed of an AEEA spacer, a γ‐glutamate linker, and a C20 fatty diacid moiety, which promotes reversible noncovalent binding to serum albumin. Noncanonical residues: Aib, α‐aminoisobutyric acid (Positions 2 and 20); αMeL, α‐methyl‐leucine (Position 13). The C‐terminus is amidated (‐NH_2_).

Multireceptor incretin agonists have emerged as a prominent class for the treatment of obesity and type 2 diabetes mellitus. Retatrutide (LY3437943) is a synthetic peptide in this class, acting as a triple agonist at the glucagon‐like peptide‐1 receptor (GLP‐1R), glucose‐dependent insulinotropic polypeptide receptor (GIPR), and glucagon receptor (GCGR) [1, 2]. Structurally, it comprises a 39‐amino‐acid sequence derived from a GIP backbone, covalently linked to a C20 fatty diacid moiety via a modified lysine residue (Figure 1). This modification promotes reversible noncovalent binding to serum albumin, resulting in an extended half‐life of approximately 6 days in humans by reducing renal filtration and proteolytic degradation [2, 3].

The pharmacological rationale for triple agonism is based on the synergistic modulation of glucose homeostasis, energy balance, and appetite regulation. Compared with mono‐ and dual‐agonist therapies including semaglutide [4] and tirzepatide [5], retatrutide has demonstrated superior metabolic efficacy in phase 2 clinical trials [6, 7], with body weight reductions of up to 24.2% at 48 weeks, and significant improvements in glycaemic control, as well as less muscle mass loss [6]. These pronounced physiological effects, including substantial reductions in fat mass, muscle maintenance, improved insulin sensitivity, and modulation of substrate utilization, highlight not only its therapeutic potential but also its appeal for misuse in weight‐sensitive and endurance‐based sports [7].

From an antidoping perspective, the emergence of such metabolic modulators presents a complex and evolving challenge. Although incretin‐based drugs are not currently included in the World Anti‐Doping Agency (WADA) Prohibited List [8], peptide hormones and their analogs fall within a category of substances historically subject to ongoing monitoring and preventive research [9, 10]. Within this context, GLP‐1 receptor agonists have attracted increasing attention in the antidoping community, with reports indicating their off‐label use among physically active individuals, reflecting concerns regarding their capacity to modulate body composition, energy substrate availability, and recovery parameters [11, 12]. The characterization of novel agonists such as retatrutide, including its pharmacokinetics and analytical detectability, is therefore relevant for doping control laboratories.

The detection of long‐acting peptide drugs in doping control samples presents considerable analytical challenges. These compounds are typically present at low concentrations relative to background biological matrix components, exhibit structural complexity with multiple ionization states, and may undergo metabolic transformations that generate diverse biotransformation products [13]. High‐resolution mass spectrometry (HRMS) platforms have become a key enabling technology in this context, supporting both targeted and nontargeted detection of intact peptides and their metabolites, as well as retrospective reanalysis of archived spectral data [9, 14]. The combination of liquid chromatography with high‐resolution accurate mass spectrometry (LC‐HRMS) allows sensitive detection, structural elucidation, and confident identification of emerging, nonregulated substances, allowing the detection and characterization of emerging substances relevant to doping control [12].

The dual GIP/GLP‐1 receptor agonist class, pioneered by compounds such as LY3298176 [15, 16], established the pharmacological precedent upon which the development of triple agonists such as retatrutide was predicated. Despite this growing clinical interest, limited information is currently available regarding the metabolic pathways and elimination profile of retatrutide in humans, beyond the early pharmacokinetic characterization conducted in healthy subjects and in patients with type 2 diabetes [17]. This gap represents a critical challenge for the establishment of an efficient antidoping strategy, as the identification of analytically suitable targets and the definition of relevant detection windows are prerequisites for the development and implementation of compliant methods. Characterization of intact peptide charge‐state distributions, albumin‐bound versus free fractions, and potential proteolytic cleavage products is essential for establishing reliable screening and confirmation strategies [18].

Here, we describe the development of a targeted LC‐HRMS method for retatrutide detection and investigate its concentration‐time profile in human biological matrices (namely human plasma and urine), with particular focus on detection window characterization relevant to antidoping testing. These findings may support the development of analytical strategies for the detection of this emerging class of long‐acting peptide therapeutics.

## 2 Materials and Methods

### 2.1 Chemicals and Reagents

Retatrutide (LY3437943; purity ≥ 95%) was obtained from Ozel Form (Istanbul, Turkey). As the volunteers independently obtained and self‐administered the substance, an aliquot of the administered material was analyzed in‐house by high‐resolution mass spectrometry prior to the study; its identity was confirmed by the expected molecular formula and characteristic fragmentation pattern, consistent with the reference structure of retatrutide. The internal standard (IS) D4‐GHRP‐4 was purchased from Anaspec (Fremont, CA, USA). Dimethyl sulfoxide (DMSO), methanol, acetonitrile, and water (all LC–MS grade), as well as formic acid (≥ 98%), were obtained from Sigma‐Aldrich (St. Louis, MO, USA). Solid‐phase extraction cartridges Oasis HLB (60 mg, 3 mL) were obtained from Waters (Milford, MA, USA) and Strata WCX (30 mg, 3 mL) from Phenomenex (Torrance, CA, USA). Blank human plasma (lithium heparin) and blank human urine were obtained from healthy volunteers with no history of drug use within 30 days prior to donation and confirmed negative for the target analyte by routine antidoping screening (including peptide hormones and small‐molecule drug classes). The present method was developed specifically for the detection of retatrutide; related GLP‐1 receptor agonists such as semaglutide and tirzepatide were not included in the current target list, although the workflow is readily extensible to these analogs in future multianalyte applications.

Although isotopically labeled structural analogs of retatrutide are not commercially available, D4‐GHRP‐4 was selected as a surrogate IS based on its features as a synthetic peptide subject to analogous solid‐phase extraction behavior under the applied conditions.

### 2.2 Sample Preparation

#### 2.2.1 Plasma

Plasma samples (500 μL), obtained from volunteers following retatrutide administration, spiked with 10 μL of internal standard solution (D4‐GHRP‐4, at a final concentration of 100 ng/mL), were transferred to 2 mL polypropylene tubes. Formic acid (50 μL, concentrated) was added, and the mixture was vortex‐mixed and incubated at 60°C for 10 min to promote disruption of the high‐affinity noncovalent interaction between the C20 fatty diacid moiety of retatrutide and serum albumin binding sites prior to protein precipitation [3, 19, 20]. Without this acid‐thermal pretreatment step, analyte recovery was insufficient for reliable detection. Subsequently, 1.5 mL of ice‐cold acetonitrile was added to induce protein precipitation. The samples were vortex‐mixed and centrifuged at 15000 × g for 10 min at 4°C. The resulting supernatant was transferred to a clean polypropylene tube and evaporated to dryness under vacuum (35°C). The dried residue was reconstituted in 3 mL of 0.1% formic acid in water and subjected to solid‐phase extraction (SPE) as described below.

#### 2.2.2 Urine

Urine samples (5 mL) were transferred to polypropylene tubes, spiked with 50 μL of internal standard solution (D4‐GHRP‐4, at a final concentration of 100 ng/mL), and vortex‐mixed for 30 s prior to direct loading onto the SPE cartridges described below, without prior protein precipitation.

To maximize the probability of detecting retatrutide‐related species across the full polarity spectrum, four complementary sample preparation strategies were applied to urine samples in parallel: (i) Oasis HLB SPE, performed under the same conditions used for plasma and described in Section 2.3 (common to both matrices); (ii) weak cation‐exchange SPE (Strata WCX, 30 mg, 3 mL; Phenomenex), conditioned with methanol and water, washed sequentially with 0.1% formic acid and 5% methanol in 0.1% formic acid, and eluted with 5% formic acid in methanol; (iii) dilute‐and‐shoot, in which urine was diluted 1:5 (v/v) with 0.1% formic acid in water and injected directly; and (iv) direct urine injection without sample preparation. All four strategies were applied to samples from all volunteers using the LC‐HRMS conditions described above.

### 2.3 Solid‐Phase Extraction (SPE) Common to Plasma and Urine

Sample clean‐up was performed using Oasis HLB cartridges (60 mg, 3 mL; Waters, Milford, MA, USA). Cartridges were conditioned sequentially with 2 mL of methanol and 2 mL of 0.1% formic acid in water. Samples were loaded at a flow rate of no more than 1 mL/min. Cartridges were subsequently washed with 2 mL of 0.1% formic acid in water, followed by 2 mL of 5% methanol in 0.1% formic acid. Analytes were eluted with 2 mL of 80% methanol in water (v/v). Eluates were evaporated to dryness under vacuum (35°C) and reconstituted in 70:30 (v/v) water–methanol containing 0.1% formic acid (v/v), vortex‐mixed for 30 s, and transferred to autosampler vials prior to LC–HRMS analysis.

### 2.4 Instrumentation and Analytical Conditions

Chromatographic separation was performed on a Thermo Scientific Ultimate 3000 UHPLC system (Thermo Fisher Scientific, Bremen, Germany) equipped with an ACE UltraCore 2.5 SuperC18 column (50 × 2.1 mm, 2.5 μm; Advanced Chromatography Technologies, Aberdeen, UK), maintained at 40°C. The mobile phases consisted of (A) water with 0.1% formic acid (v/v) and 1% DMSO (v/v), and (B) acetonitrile with 0.1% formic acid (v/v) and 1% DMSO (v/v). A proportion of 1% (v/v) DMSO was added to both mobile phases to improve chromatographic peak shape and reduce nonspecific adsorption of the hydrophobic lipidated analyte to the metallic surfaces of the LC system, as previously described for fatty acid‐modified peptides [3, 6]. The flow rate was 0.30 mL/min and the injection volume was 10 μL. A binary gradient from 5% to 99% B (0.5–14.0 min) was applied, followed by re‐equilibration at initial conditions (14.1–20.0 min), resulting in a total run time of 20 min.

Mass spectrometric detection was performed on a Q Exactive Plus Orbitrap mass spectrometer (Thermo Fisher Scientific, Bremen, Germany) operated in positive electrospray ionization (ESI+) mode. Full MS scans were acquired over *m*/*z* 600–1700 at a resolution of 70 000 FWHM (AGC target: 3 × 10^6^; maximum injection time: 100 ms), followed by data‐dependent MS/MS acquisition (ddMS2 mode: Top3; resolution 17 500 FWHM at the nominal *m*/*z* 200; normalized collision energy (NCE): 20). Targeted‐SIM (t‐SIM) acquisition mode was conducted at a resolution of 70 000 FWHM (AGC target: 5 × 10^4^; maximum injection time: 15 ms) with an isolation window of 4.0 *m*/*z*. For confirmatory analysis, parallel reaction monitoring (PRM) was conducted at an NCE of 20, monitoring structurally diagnostic b‐ and y‐series fragment ions. Targeted acquisition parameters, including precursor ion selection and characteristic fragment ions, are summarized in Table 1. The mass spectrometer was calibrated daily using the manufacturer's calibration solution (Thermo Fisher Scientific) in positive ionization mode to ensure mass accuracy throughout the analytical sequence.

**TABLE 1:** Optimized targeted LC‐HRMS acquisition parameters for retatrutide. The [M + 3H]^3+^ charge state was selected based on the predominant ion observed in full MS spectra of the reference standard. Fragment ion assignments are based on in silico prediction and experimental confirmation by PRM.

Compound	Charge state	*m*/*z*	Fragment ions—PRM (*m*/*z*)	NCE
Retatrutide (LY3437943)	[M + 3H]^3+^	1578.1648	396.2228 (y_4_); 909.4391 (y_11_); 2169.1286 (b_35_^2+^)	20

### 2.5 Method Validation

Method validation was conducted in accordance with the WADA International Standard for Laboratories (ISL 2021) [21]. Selectivity was evaluated through the analysis of 10 different blank plasma and urine samples to confirm the absence of interfering signals at both the retention times and *m*/*z* values of the target analyte. Reliability was assessed using 10 independently prepared samples, each fortified to 200 ng/mL in each matrix [18]. Analyte concentrations in authentic samples were estimated by single‐point calibration against a calibrator prepared by fortifying blank matrix with retatrutide at 50 ng/mL, which was processed and analyzed alongside the study samples in each analytical batch to monitor method performance.

The limit of detection (LOD) was determined by spiking retatrutide into 10 different samples per matrix across six concentration levels (1–200 ng/mL). A rate of at least ≥ 95% of detectability was applied as the acceptance criterion following LC‐HRMS/MS analysis. LOD values were additionally estimated using logistic regression modeling of detection probability as described by Garrido et al. [22] Robustness was assessed by analyzing 10 fortified samples under modified experimental conditions, including minor variations in reconstitution volume and SPE wash volume. Carryover was evaluated by injecting blank samples immediately following high‐concentration samples (400 ng/mL). The stability of processed samples was investigated by reanalysis after storage in the autosampler at 10°C for 72 h. Finally, extraction recovery was determined at 100 ng/mL by comparing the peak areas obtained from samples fortified prior to extraction with those from samples fortified after the complete sample preparation procedure. Matrix effects (ion suppression or enhancement) were not systematically evaluated in the present study; this is acknowledged as a limitation, and their assessment following established approaches [23] is recommended for full quantitative validation of the method.

### 2.6 Elimination Study

An elimination study was conducted with four healthy volunteers (*n* = 4; 2 males, 2 females; mean age 36.8 ± 7.4 years) to characterize the concentration‐time profiles of retatrutide in plasma and urine after administration. No relevant co‐medications were reported during the study period.

The study monitored voluntary donors following independent self‐administration of two 2‐mg subcutaneous doses of retatrutide into the abdominal subcutaneous tissue on Days 1 and 7 prior to urine and plasma collection. The reported exposure is consistent with early dose‐escalation regimens described in retatrutide clinical development programs, in which treatment initiation at 2 mg once weekly has been described before escalation toward higher maintenance doses [24, 25]. Biological samples (lithium heparin plasma and urine samples) were collected prior to the first administration (0 h) and at 2, 24, 48, 72, 96, 120, 144, 168, 336, 504, 672, and 1344 h postadministration (Days 1, 2, 3, 4, 5, 6, 7, 8, 14, 21, 28, and 56, respectively). Plasma samples were processed immediately upon collection and stored at −80°C until analysis. Urine samples were collected as a single complete (total) void, aliquoted, and stored at −20°C.

The study protocol was approved by the local ethics committee (approval no. 95572126.2.0000.0268), and all participants provided written informed consent prior to inclusion. The study was conducted in accordance with the principles of the Declaration of Helsinki (2013 revision).

## 3 Results

### 3.1 Method Validation

Retatrutide was monitored as the [M + 3H]^3+^ precursor ion at *m*/*z* 1578.16 using targeted‐SIM (t‐SIM) acquisition. Selectivity was confirmed by the absence of interfering signals at the retention time and corresponding *m*/*z* values of retatrutide in all blank samples analyzed (*n* = 10 per matrix), confirming method specificity in both plasma and urine. No carryover was observed, as blank injections performed immediately after high‐concentration samples produced no detectable signal for the target analyte.

**FIGURE 2:**
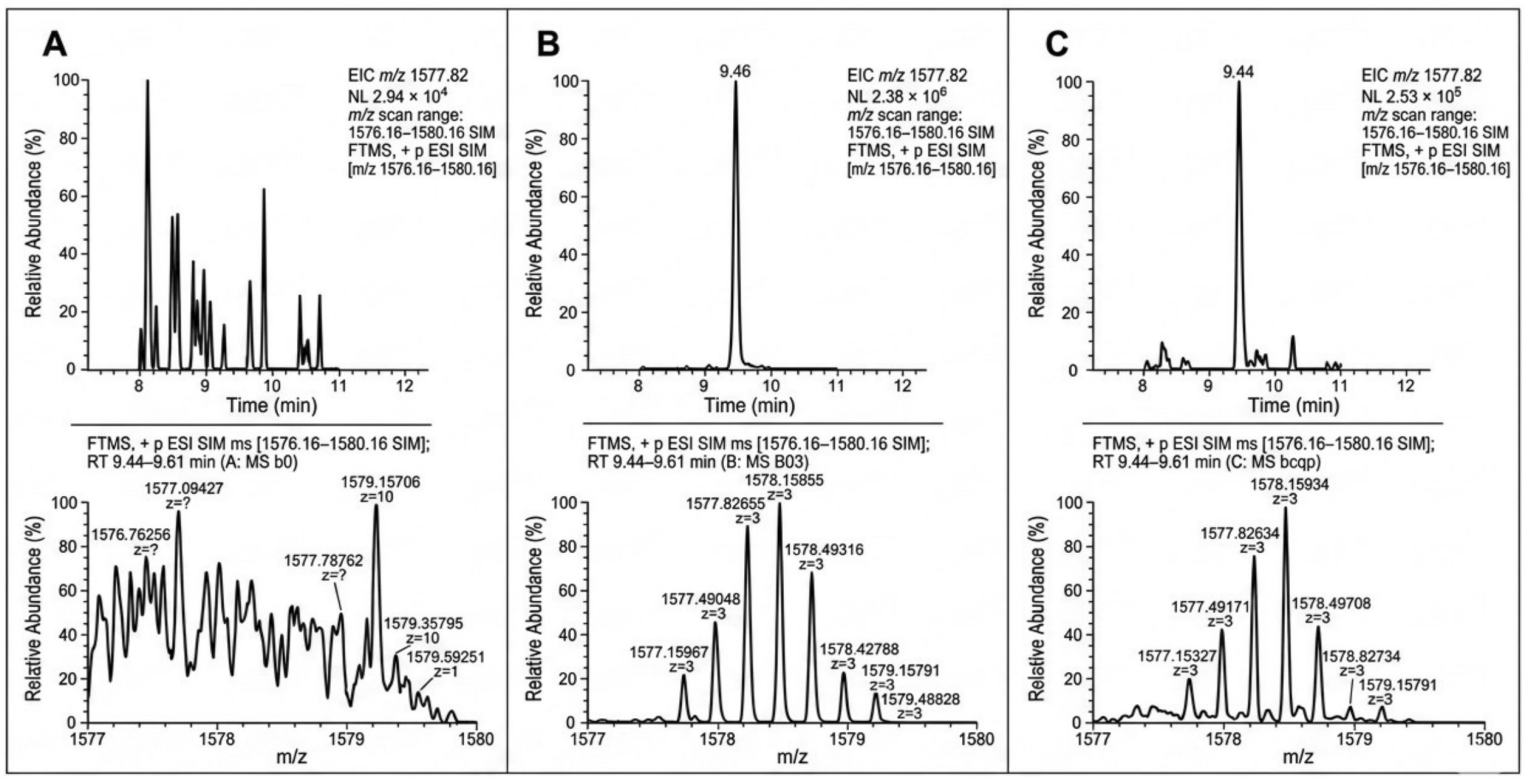
Representative extracted ion chromatograms and corresponding mass spectra of retatrutide ([M + 3H]^3+^, t‐SIM) in (A) blank plasma (negative control), (B) plasma from a volunteer collected 24 h after the first administration, and (C) blank plasma fortified at 50 ng/mL (positive control). For each sample, the upper panel shows the extracted ion chromatogram (EIC) of the retatrutide [M + 3H]^3+^ precursor and the lower panel shows the corresponding SIM mass spectrum averaged over the peak apex (RT 9.44–9.61 min), displaying the characteristic isotopic envelope of the triply charged ion. The multiple low‐intensity signals in the blank chromatogram (A) correspond to background noise (note the low intensity scale, NL 2.94 × 10^4^) and do not represent a defined analyte peak, whereas the postdose (B) and spiked (C) samples show a single well‐resolved peak at the expected retention time.

**FIGURE 3:**
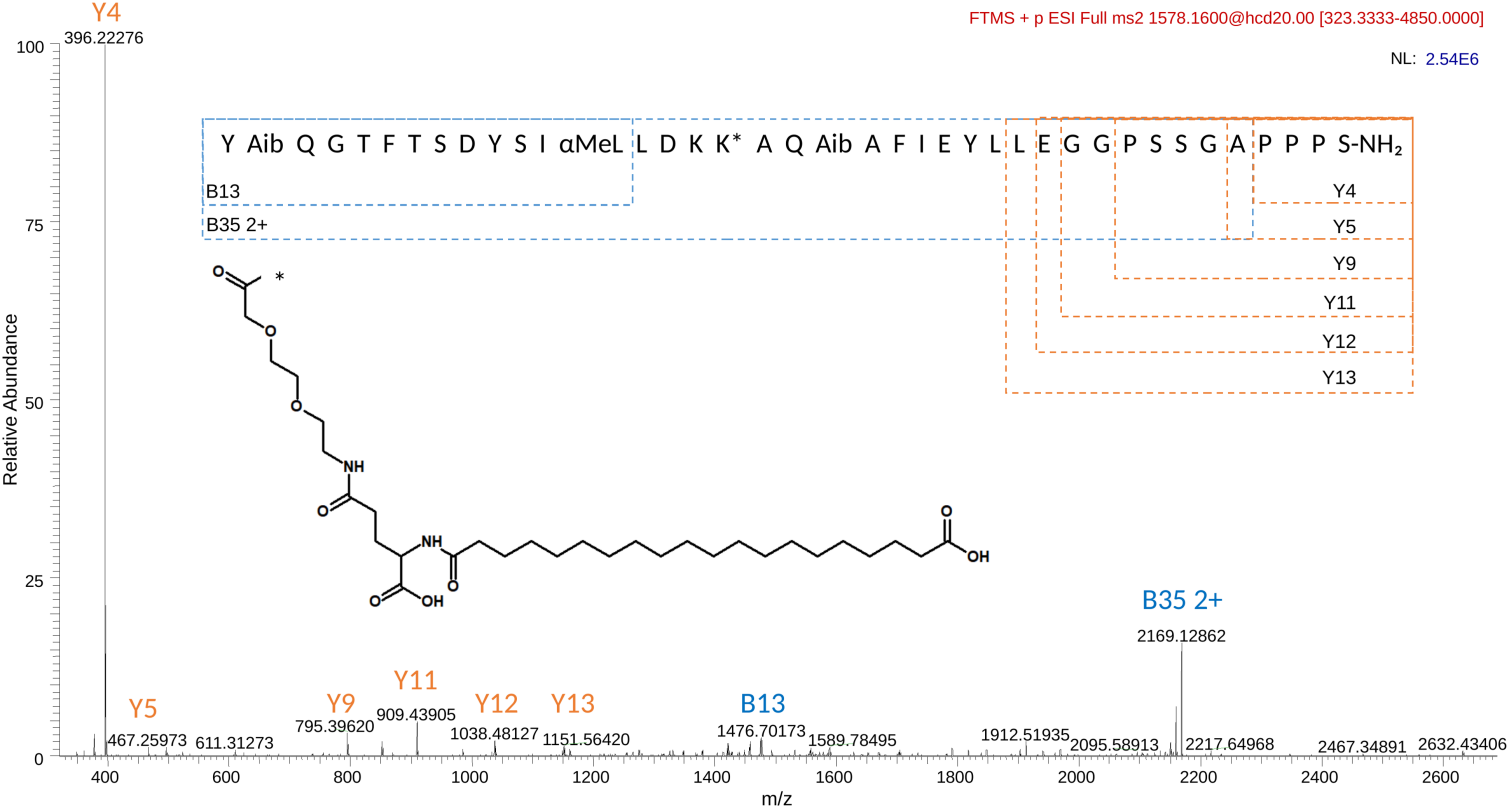
Annotated amino acid sequence of retatrutide (39 residues) showing the eight diagnostic fragment ions identified in the PRM spectrum (precursor [M + 3H]^3+^ at *m*/*z* 1578.16; HCD, NCE 20). Dashed vertical lines indicate the backbone cleavage sites yielding b_13_, y_13_, y_12_, y_11_, y_9_, y_5_, y_4_ (base peak), and b_35_^2+^; y_4_ and b_35_^2+^ originate from the same cleavage event between residues Pro35 and Pro36. K17* denotes the modified lysine residue carrying the AEEA‐γGlu‐C20‐fatty diacid moiety, which is retained in the b_35_^2+^ fragment and thus serves as a structurally diagnostic marker of the intact analog. Aib, α‐aminoisobutyric acid; αMeL, α‐methyl‐leucine.

Method reliability was confirmed by successful detection of retatrutide in all fortified samples (10/10) at 200 ng/mL in both matrices. Robustness was further evaluated under slightly modified experimental conditions, including small variations in reconstitution volume and SPE wash volume. These changes did not significantly affect analyte detection or identification, and the WADA identification criteria remained fulfilled under all modified experimental conditions, indicating the method's stability. Logistic regression modeling of detection probability yielded estimated LOD values of 6.14 ng/mL in plasma and 2.44 ng/mL in urine. The complete validation results are summarized in Table 2. Stability assessment confirmed that processed samples stored in the autosampler at 10°C remained stable for up to 72 h without measurable signal loss. Representative extracted ion chromatograms of blank plasma, postdose plasma, and fortified plasma demonstrating the selectivity of the method are shown in Figure 2. The identity of retatrutide was confirmed by acquisition of product ion spectra in PRM mode. In authentic postadministration samples, mass accuracies better than 5 ppm were obtained both for the precursor ion and for the diagnostic product ions (e.g., the b_35_^2+^ fragment was measured at *m*/*z* 2169.1286, corresponding to a deviation below 1 ppm from the theoretical value). Although eight b‐ and y‐series fragment ions were observed (Figure 3), confirmation of analyte identity in each positive sample was based on the three most intense and structurally diagnostic product ions (y_4_, y_11_, and b_35_^2+^), which consistently fulfilled the WADA TD2023IDCR identification criteria; the remaining fragment ions provided additional supporting evidence.

**TABLE 2:** Summary of method validation results for retatrutide in human plasma and urine.

Parameter	Plasma	Urine
Selectivity	No interference (*n* = 10)	No interference (*n* = 10)
Reliability	10/10 at 200 ng/mL	10/10 at 200 ng/mL
LOD—logistic regression	6.14 ng/mL	2.44 ng/mL
Robustness	No significant variation	No significant variation
Carryover	Not detected	Not detected
Processed sample stability	Stable (72 h, 10°C)	Stable (72 h, 10°C)
Extraction recovery (100 ng/mL)	63.8%	31.4%

### 3.2 Elimination Study

Retatrutide was successfully detected in plasma samples from all four volunteers following subcutaneous administration. No detectable signal was observed at baseline (0 h), indicating the absence of endogenous background or interference, in alignment with the validation results. A representative extracted ion chromatogram (EIC) of retatrutide ([M + 3H]^3+^, t‐SIM) obtained from a plasma sample collected at 24 h after administration is shown in Figure 2 (panel B). The chromatogram exhibits a single well‐resolved, interference‐free peak at the expected retention time, in contrast to the blank sample (panel A), in which only low‐intensity background signals were observed.

**FIGURE 4:**
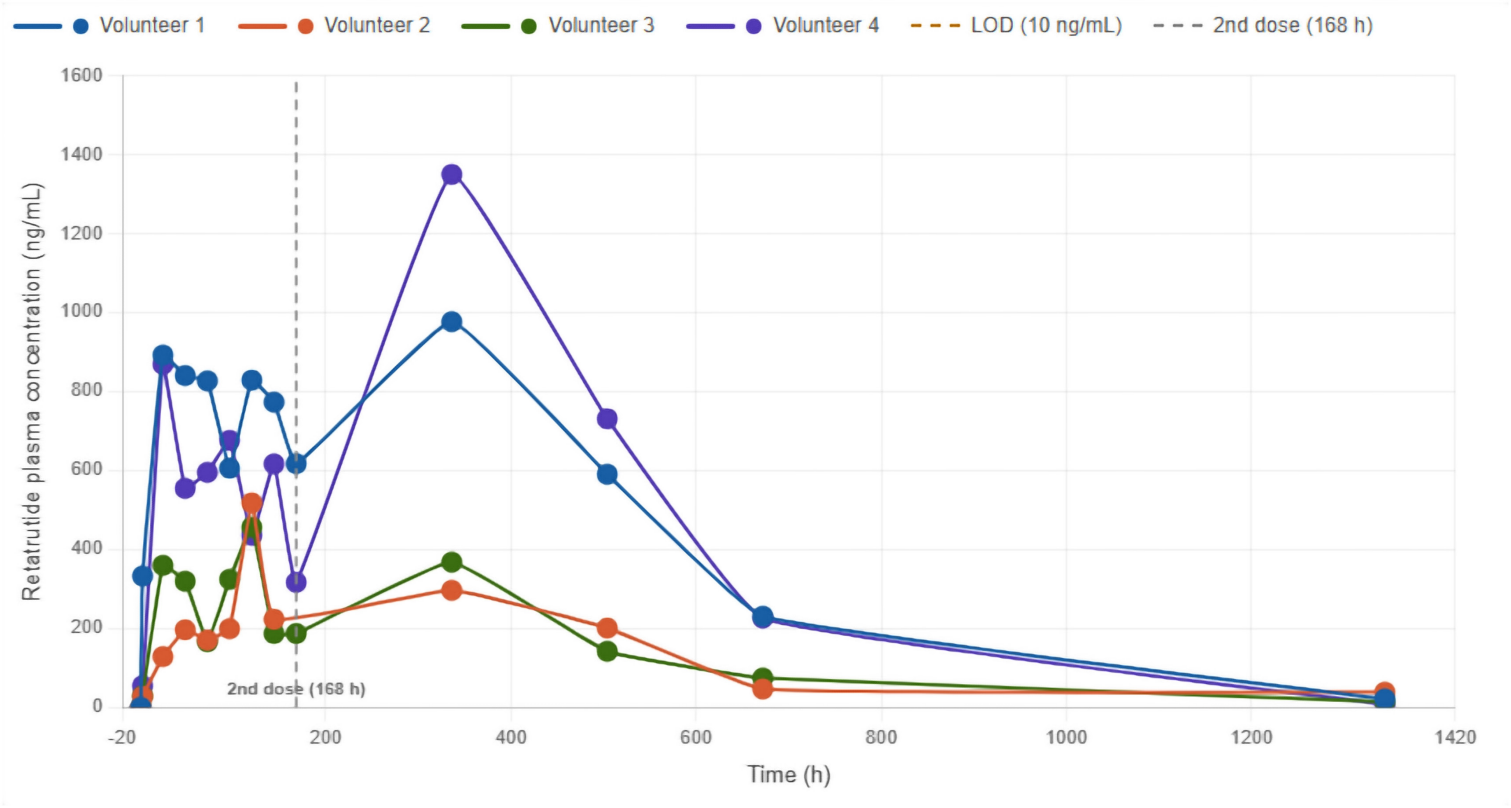
Individual plasma concentration–time profiles of retatrutide in four healthy volunteers (*n* = 4) following two subcutaneous administrations of 2 mg on Day 1 (0 h) and Day 7 (168 h, dashed vertical line). Data points represent estimated plasma concentrations (ng/mL) determined by LC‐HRMS using external calibration. The sample from Volunteer 2 at 168 h was unavailable; the connecting line interpolates between the adjacent time points (144 h and 336 h).

The individual plasma concentration–time profiles of retatrutide are presented in Figure 4. Detectable concentrations of retatrutide were observed at 2 h postadministration in all volunteers, with peak plasma levels typically occurring between 24 and 48 h after the first dose and showing marked interindividual variability. Elevated concentrations persisted up to 144 h. A secondary increase in plasma levels was observed in multiple participants at 336 h (i.e., 14 days post–first dose, 7 days after the second administration on Day 7). This secondary elevation is consistent with the contribution of the second subcutaneous dose and with the prolonged depot‐like subcutaneous absorption profile commonly associated with albumin‐binding lipidated peptides. Marked interindividual variability was observed in peak concentrations (*C*_max_ at 24 h: 128.6–892.3 ng/mL across volunteers), consistent with known variability in subcutaneous absorption, body composition, and individual albumin‐binding capacity.

Retatrutide remained detectable in all four volunteers at 672 h (28 days) after the first dose, corresponding to 504 h (21 days) after the second dose, with plasma concentrations ranging from 46.4 to 230.8 ng/mL. At the final sampling point at 1344 h (56 days) after the first dose, corresponding to 1176 h (49 days) after the second dose, all participants still had detectable analyte concentrations of 7.1–38.4 ng/mL. The corresponding quantitative data are summarized in Table 3.

**TABLE 3:** Individual plasma concentrations (ng/mL) of retatrutide in four volunteers following two subcutaneous administrations (2 mg on Days 1 and 7, corresponding to 0 and 168 h). Values are expressed as estimated concentrations based on external calibration. ND, not detected above the LOD; —, sample not available.

Time (h)	Volunteer 1	Volunteer 2	Volunteer 3	Volunteer 4
0	ND	ND	ND	ND
2	332.7	27.7	29.0	54.8
24	892.3	128.6	359.7	869.0
48	840.4	196.4	319.4	554.8
72	826.9	170.5	166.1	595.2
96	605.8	199.1	324.2	676.2
120	828.8	517.9	456.5	435.7
144	773.1	223.2	187.1	616.7
168^a^	617.3	—	187.1	316.7
336	976.9^b^	296.4^b^	367.7^b^	1350.0^b^
504	590.4	200.9	141.9	731.0
672	230.8	46.4	74.2	226.2
1344	21.2	38.4	14.5	7.1

^a^ Second dose (2 mg subcutaneous) administered at this time point.

^b^ Secondary elevation consistent with cumulative pharmacokinetic contribution of the second dose administered at 168 h (Day 7).

**FIGURE 5:**
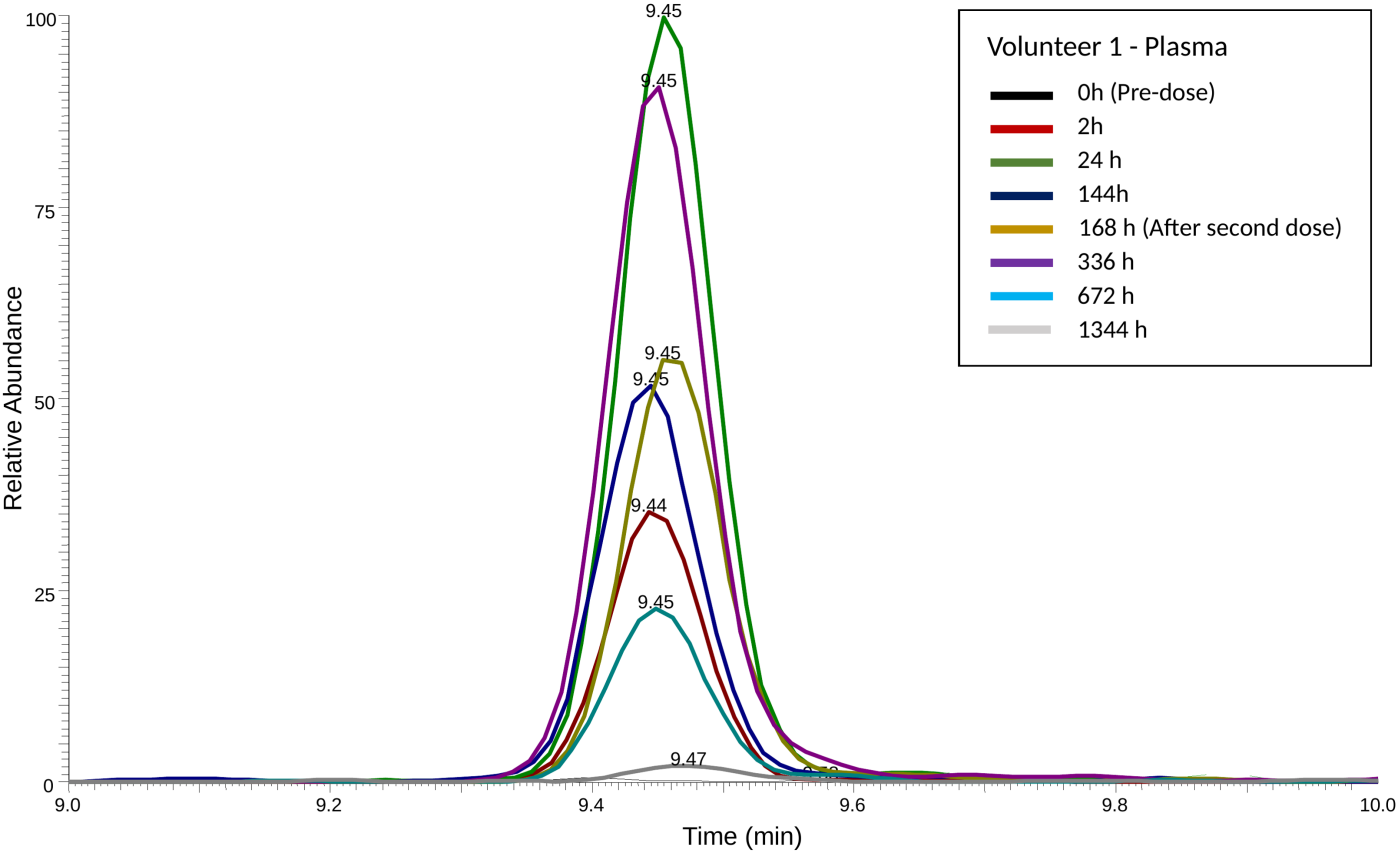
Overlaid extracted ion chromatograms (EICs) of retatrutide ([M + 3H]^3+^, *m*/*z* 1578.16 ± 5 ppm) in plasma samples from a single representative volunteer (Volunteer 1) collected at eight postadministration time points across the 56‐day observation period: 0 h (predose), 2 h, 24 h, 144 h, 168 h (after second dose), 336 h, 672 h, and 1344 h. All chromatograms were acquired by t‐SIM under identical LC‐HRMS conditions. The consistent retention time of 9.45 min across all time points supports the identity of the detected species.

To provide direct chromatographic evidence of retatrutide persistence in plasma throughout the observation period, extracted ion chromatograms acquired from a volunteer (Volunteer 1) are overlaid in Figure 5. The consistent retention time of 9.45 min across all sampling points, combined with the reproducible peak shape, supports that the species detected at 1344 h corresponds to intact retatrutide and not to a co‐eluting interference. This visual evidence supports the extended plasma detection window of 56 days reported in this study.

In contrast, neither intact retatrutide nor any metabolite‐like signal corresponding to expected peptide cleavage products was detected in the urine samples from any of the volunteers at any time points throughout the 56‐day collection period.

The absence of retatrutide‐derived signals in urine was consistent across all four sample preparation strategies evaluated, including direct urine injection, dilute‐and‐shoot, reversed‐phase HLB SPE, and weak cation‐exchange SPE (Strata WCX). This convergent negative result supports that the absence of urinary markers is not attributable to matrix‐dependent extraction losses or selectivity limitations of a single sample preparation approach.

## 4 Discussion

The present study demonstrates the detection of intact retatrutide in human plasma by LC‐HRMS, with a detection window extending to at least 1344 h (56 days) following subcutaneous administration. To our knowledge, this is the first report characterizing the concentration‐time profile of retatrutide in human biological fluids in an antidoping context. Although further studies are needed to investigate the possible formation of metabolites, the prolonged detectability of the intact molecule in plasma suggests a high degree of in vivo structural stability and supports the use of the parent compound as the primary analytical target for doping control applications. It is important to clarify what is meant here by an antidoping context. Under the current WADA regulatory framework, doping control for this class of substances relies on urine as the routinely collected matrix, and blood‐based analysis of incretin receptor agonists is not presently part of routine antidoping practice. The present findings, however, point to a fundamental mismatch between this practice and the analytical behavior of retatrutide, which was undetectable in urine yet persistently measurable in plasma for up to 56 days. In this specific sense, the antidoping relevance of the study lies not in proposing an immediately applicable urinary test, but in providing evidence that the detection of retatrutide and, by extension, other long‐acting lipidated peptide agonists would require the adoption of blood‐based matrices.

The plasma concentration–time profiles showed sustained systemic exposure and a nonmonotonic pattern, including secondary increases in concentration observed mainly around 336 h post–first dose. This secondary increase is attributable to the cumulative pharmacokinetic contribution of the second dose administered at 168 h (Day 7), superimposed on residual systemic levels from the first dose, and is consistent with depot‐like subcutaneous absorption behavior. A central structural determinant of this profile is the C20 fatty diacid moiety covalently attached via a modified lysine residue, which promotes reversible noncovalent binding to circulating serum albumin [3]. This interaction is known to reduce renal filtration and slow proteolytic degradation, thereby extending systemic half‐life and delaying elimination, a strategy commonly employed in long‐acting peptide analogs [6, 26]. The marked interindividual variability in peak concentrations (*C*_max_ at 24 h: 128.6–892.3 ng/mL) is consistent with differences in subcutaneous absorption, body composition, and albumin binding affinity among individuals, in line with the dose‐proportional pharmacokinetic behavior previously reported for retatrutide in repeated‐dose phase 1b studies [17]. A terminal elimination half‐life was estimated from the log‐linear decay phase following the concentration peak observed after the second dose (*t* > 336 h). In the three volunteers exhibiting well‐fitted mono‐exponential elimination (*R* ^2^ > 0.96), the estimated terminal half‐life ranged from approximately 5.5 to 9.6 days (mean ≈ 7.5 days), which is broadly consistent with, though slightly longer than, the terminal half‐life of approximately 6 days reported in phase 1 pharmacokinetic studies [2]. The marginally longer values observed here may reflect the extended sampling window of the present study, which captured a slower terminal elimination phase, together with the limited cohort size and the semi‐quantitative nature of the single‐point calibration. One volunteer exhibited an irregular terminal decay that did not conform to mono‐exponential elimination, precluding reliable half‐life estimation and underscoring the interindividual variability expected in a small cohort.

A central finding of this study is the absence of detectable intact retatrutide, as well as any metabolite‐like signals consistent with expected peptide cleavage products, in urine samples collected over the 56‐day follow‐up period. This result is particularly noteworthy when compared with structurally related compounds. For example, tirzepatide has been reported to produce detectable metabolite‐derived species in urine in human ADME studies [24]. To our knowledge, no dedicated human ADME study for retatrutide has been published to date, and the present work therefore provides the first experimental assessment of the urinary fate of the compound in humans. Despite applying extraction strategies based on established methodologies for related compounds, including comparable SPE conditions and elution protocols, no analogous urinary markers for retatrutide were identified. Notably, post hoc analyses of phase 2 retatrutide trials have reported only modest changes in urinary albumin‐to‐creatinine ratio and estimated glomerular filtration rate [27], supporting the view that the renal handling of retatrutide is largely uncoupled from the appearance of analytically meaningful drug‐related species in urine.

An additional methodological aspect concerns the acid‐thermal pretreatment step incorporated into the plasma sample preparation workflow. The combination of concentrated formic acid and incubation at 60°C for 10 min proved necessary to achieve adequate analyte recovery [24]. The requirement for a more energetically demanding disruption strategy in the present study is consistent with the higher albumin affinity conferred by the C20 fatty diacid moiety of retatrutide, compared with the C18 moiety of tirzepatide, as stronger albumin binding is expected to require more aggressive conditions to release the protein‐bound fraction [3, 4, 19]. The use of formic acid to release protein‐bound hydrophobic analytes prior to organic precipitation has been described in untargeted plasma metabolomics [20]. However, to our knowledge, its combination with thermal treatment has not been previously described for lipidated therapeutic peptides in a doping control context. From a practical standpoint, these findings suggest that conventional protein precipitation protocols, when applied without prior acid‐thermal treatment, may result in reduced recovery or even false‐negative results for retatrutide and potentially other GLP‐1 analogs with similar or greater albumin affinity.

To investigate whether potential retatrutide metabolites might be present below the detection capability of the primary method, a systematic targeted search was performed using an in silico‐derived list of theoretical metabolite masses, guided by the established metabolic pathways of structurally related lipidated GLP‐1 analogs. For semaglutide, the two primary urinary metabolites identified in humans (U6 and U7) consist of the free Lys26 amino acid bound to the linker with butyric (C4) or hexanoic (C6) di‐acid side chains, formed through complete proteolytic cleavage of the peptide backbone and sequential beta‐oxidation of the C18 fatty diacid [28]. By structural analogy, the equivalent targets for retatrutide were defined as: (i) intact‐backbone beta‐oxidation products of the C20 fatty diacid (B‐series: M0‐C18 through M0‐C4, corresponding to one through eight beta‐oxidation cycles); (ii) modified lysine (K*) fragments retaining the fatty diacid at successively shorter chain lengths (K*‐C18 through K*‐C4, the direct structural equivalents of semaglutide U7 and U6); (iii) proteolytic backbone cleavage fragments retaining the K* moiety (F‐series, based on predicted endoprotease cleavage sites); and (iv) 3‐hydroxyacyl intermediates of the beta‐oxidation pathway (OH‐series). High‐resolution mass matching was performed using a 10‐ppm tolerance across multiple charge states. This was a deliberately conservative tolerance, as the instrument routinely achieves mass accuracies below 5 ppm. It was chosen to maximize the sensitivity of the search and minimize the risk of overlooking low‐abundance candidates. No metabolite signals were detected in authentic postdose urine or plasma samples beyond the intact parent compound (Table S1). This absence may be attributed to a critical structural distinction: while semaglutide and tirzepatide require seven sequential beta‐oxidation cycles to reduce their C18 fatty diacid to the terminal C4 fragment detectable in urine, the C20 chain of retatrutide requires an additional eighth cycle to reach the analogous K*‐C4 species (e.g., theoretical *m*/*z* 521.2454 for the [M + H]^+^ ion [neutral monoisotopic mass 520.2381 Da; Table S1]). Coupled with the superior albumin binding of retatrutide and prolonged half‐life, this additional metabolic step likely results in urinary concentrations remaining below detection capabilities or a shift toward biliary and fecal excretion route [28].

The absence of urinary signals was observed with all four sample preparation approaches, which covered complementary selectivity windows. While Oasis HLB SPE preferentially retains moderately to highly hydrophobic analytes, consistent with the lipidated character of retatrutide and its larger beta‐oxidation intermediates, weak cation‐exchange SPE was additionally applied to capture more polar, cationic peptide fragments that may be underrepresented in reversed‐phase extraction. Dilute‐and‐shoot and direct urine injection were also evaluated to eliminate the possibility that metabolites were being lost during sample preparation. None of these approaches yielded detectable signals for any retatrutide‐related species, supporting the view that the absence of urinary markers reflects a genuine pharmacokinetic phenomenon rather than a methodological limitation. Together, these approaches reduced the likelihood that the absence of urinary signals was caused by a limitation of a single sample preparation procedure. This interpretation is mechanistically consistent with the established pharmacokinetics of albumin‐binding fatty acid‐conjugated peptides, which are predominantly eliminated through hepatic and proteasomal pathways rather than renal filtration of the intact high‐molecular‐weight molecule [3, 22, 23].

From an antidoping perspective, these findings highlight a potential limitation of urine‐based detection for retatrutide. Urine remains the primary matrix for routine doping control due to its noninvasive collection and well‐established analytical frameworks [9]. However, the present data, together with previous observations for tirzepatide [24], suggest that long‐acting incretin‐based peptides may be poorly suited for detection in urine, not because of analytical limitations, but due to their intrinsic pharmacokinetic behavior. These findings do not exclude the possibility that urinary metabolites may be useful analytical targets. However, under the conditions investigated here, plasma provided more consistent detection of intact retatrutide. In this study, plasma analysis enabled detection and identification of intact retatrutide for at least 56 days, which exceeds the detection windows typically reported for peptide‐based doping agents in urine [9, 12]. It should be emphasized that, under the current WADA regulatory framework, urine is the matrix routinely collected for the detection of this class of substances, whereas blood‐based matrices are not yet employed for their monitoring. The present findings support the consideration of alternative matrices, such as whole blood, serum or dried blood spots (DBS), for future antidoping studies of long‐acting incretin receptor agonists. DBS may offer the advantages of a blood‐based matrix while allowing minimally invasive and logistically convenient collection. Its suitability for retatrutide detection should be evaluated in future studies. The extended detection window observed in plasma, combined with the targeted analytical approach described here (t‐SIM for initial detection, PRM for confirmatory identification), provides a solid basis for the implementation of a plasma‐based doping control method aligned with WADA identification criteria [29]. Based on the minimum plasma concentrations observed at the final sampling point (7.1–38.4 ng/mL at 1344 h), a Minimum Required Performance Level (MRPL) of approximately 5 ng/mL in plasma appears technically achievable with the current method and would provide coverage of the full detection window. The formal establishment of such an MRPL would, however, require broader interlaboratory validation and regulatory consideration within the WADA framework, including evaluation of physiological baseline levels in therapeutic contexts. Moreover, the definition of an appropriate MRPL should account not only for the terminal elimination phase but also for the multidose posology and the potential accumulation of retatrutide upon repeated administration. As therapeutic use involves progressive dose escalation over weeks to achieve clinically relevant weight loss, unlike the acute, short‐lived exposure associated with agents such as diuretics, steady‐state plasma concentrations in real‐world use may substantially exceed those observed in the present two‐dose protocol, a factor that should be incorporated into future MRPL considerations.

Several limitations of the present study should be considered. First, the small sample size (*n* = 4) limits the generalizability of the presented pharmacokinetic profiles, and greater interindividual variability may be observed in a larger population. Accordingly, the elimination profile reported here should be regarded as preliminary until confirmed in a larger cohort and under different dosing regimens. Second, the limited dosing regimen used in this study (2 × 2 mg) may not fully reflect the excretion dynamics expected at higher clinical doses (up to 12 mg/week), at which any renally excreted species, if formed, might reach higher concentrations. Third, the reported plasma concentrations should be regarded as semi‐quantitative estimates, as they were derived from single‐point calibration rather than a full calibration curve. Absolute values, particularly for samples outside the immediate range of the calibrator, should therefore be interpreted with caution. Finally, the application of untargeted metabolomic approaches may reveal trace‐level or structurally modified retatrutide‐derived species not captured by the present targeted method, and represents a relevant direction for future investigation.

## 5 Conclusions

The present study shows that intact retatrutide is consistently detected in human plasma by LC‐HRMS for at least 56 days following subcutaneous administration of two 2 mg doses, representing, to our knowledge, the longest detection window reported so far for this emerging triple incretin receptor agonist. The validated analytical method, based on t‐SIM acquisition with PRM confirmation on an Orbitrap platform, satisfies key WADA criteria for selectivity, reliability, robustness, and stability and is suitable for plasma‐based doping control applications.

In contrast, neither intact analyte nor metabolite‐derived signals were detected in urine at any time point in any volunteer, despite multiple extraction approaches. This finding, together with the observed pharmacokinetic profile characterized by prolonged albumin‐mediated exposure, suggests that renal excretion of analytically relevant retatrutide‐related species is limited under the conditions investigated.

Taken together, these findings suggest a consistent pattern for long‐acting incretin‐based lipidated peptides: extended plasma detectability combined with the absence of urinary markers constitutes a systematic challenge to conventional urine‐based antidoping strategies. Plasma appears to be a more appropriate matrix for the detection of retatrutide and potentially other structurally related compounds. Overall, the present findings provide a basis for the development of plasma‐based doping control strategies and support consideration of retatrutide in future antidoping research and monitoring strategies.

## Author Contributions

**Gabriel Reis Alves Carneiro:** conceptualization, data curation, formal analysis, methodology, investigation, validation, visualization, project administration, writing – original draft. **Isabelle Karine da Costa Nunes:** conceptualization, data curation, investigation, methodology, visualization, writing – original draft. **Gustavo Ramalho dos Santos Cardoso:** conceptualization, investigation, data curation, validation, methodology, writing – review and editing. **Paola Façanha dos Santos:** conceptualization, data curation, formal analysis, investigation, methodology. **Monica Costa Padilha:** conceptualization, funding acquisition, investigation, resources, writing – review and editing, supervision. **Fábio César Sousa Nogueira:** conceptualization, funding acquisition, investigation, resources, writing – review and editing, supervision. **Henrique Marcelo Gualberto Pereira:** conceptualization, investigation, supervision, funding acquisition, resources, writing – review and editing.

## Ethics Statement

The study was approved by the Institutional Ethics Committee (approval no. 95572126.2.0000.0268). All participants provided written informed consent prior to inclusion. The study was conducted in accordance with the principles of the Declaration of Helsinki (2013 revision).

## Conflicts of Interest

The authors declare no conflicts of interest.

## Supporting information


**Table S1:** Theoretical monoisotopic masses of putative retatrutide metabolites investigated by targeted screening.

## Data Availability

The data that support the findings of this study are available from the corresponding author upon reasonable request.
