# Supplementary material for: Detection and Excretion Profile of Retatrutide in Human Plasma and Urine by LC‐HRMS: Implications for Antidoping Analysis
Source: Rapid Commun Mass Spectrom. 2026 Sep 15;40(23):e70179. doi: 10.1002/rcm.70179 (PMC13578828; doi:10.1002/rcm.70179)
Supplement: Supplementary file 1 [file RCM-40-e70179-s001.docx]

**Table S1. Theoretical monoisotopic masses of putative retatrutide metabolites investigated by targeted screening**

Candidate metabolites were derived by analogy to the established metabolic pathways of lipidated GLP-1 analogues (e.g. semaglutide). Theoretical m/z values were cross-matched against post-dose plasma and urine features at ±10 ppm across charge states z = 1–4.

| Series | ID | Structural description | Neutral mono. mass (Da) | Main ion m/z | Detected |
| --- | --- | --- | --- | --- | --- |
| β-oxidation | **B-01** | M0-C18 (1× β-ox) | 4700.4405 | 1567.8208 ([M+3H]³⁺) | n.d. |
| β-oxidation | **B-02** | M0-C16 (2× β-ox) | 4672.4092 | 1558.4770 ([M+3H]³⁺) | n.d. |
| β-oxidation | **B-03** | M0-C14 (3× β-ox) | 4644.3779 | 1549.1332 ([M+3H]³⁺) | n.d. |
| β-oxidation | **B-04** | M0-C12 (4× β-ox) | 4616.3466 | 1539.7895 ([M+3H]³⁺) | n.d. |
| β-oxidation | **B-05** | M0-C10 (5× β-ox) | 4588.3153 | 1530.4457 ([M+3H]³⁺) | n.d. |
| β-oxidation | **B-06** | M0-C8 (6× β-ox) | 4560.2840 | 1521.1019 ([M+3H]³⁺) | n.d. |
| β-oxidation | **B-07** | M0-C6 (7× β-ox) | 4532.2527 | 1511.7582 ([M+3H]³⁺) | n.d. |
| β-oxidation | **B-08** | M0-C4 (8× β-ox) | 4504.2214 | 1502.4144 ([M+3H]³⁺) | n.d. |
| K*-fragment | **K*-C18** | Lys17-AEEA-γGlu-C18 diacid | 716.4572 | 717.4645 ([M+H]⁺) | n.d. |
| K*-fragment | **K*-C16** | Lys17-AEEA-γGlu-C16 diacid | 688.4259 | 689.4332 ([M+H]⁺) | n.d. |
| K*-fragment | **K*-C14** | Lys17-AEEA-γGlu-C14 diacid | 660.3946 | 661.4019 ([M+H]⁺) | n.d. |
| K*-fragment | **K*-C12** | Lys17-AEEA-γGlu-C12 diacid | 632.3633 | 633.3706 ([M+H]⁺) | n.d. |
| K*-fragment | **K*-C10** | Lys17-AEEA-γGlu-C10 diacid | 604.3320 | 605.3393 ([M+H]⁺) | n.d. |
| K*-fragment | **K*-C8** | Lys17-AEEA-γGlu-C8 diacid | 576.3007 | 577.3080 ([M+H]⁺) | n.d. |
| K*-fragment | **K*-C6** | Lys17-AEEA-γGlu-C6 diacid | 548.2694 | 549.2767 ([M+H]⁺) | n.d. |
| K*-fragment | **K*-C4** | Lys17-AEEA-γGlu-C4 diacid | 520.2381 | 521.2454 ([M+H]⁺) | n.d. |

*n.d., not detected.*
